# Immune checkpoint inhibitors in hepatocellular carcinoma therapy: resistance mechanisms, liver transplantation challenges and management strategies

**DOI:** 10.20517/cdr.2025.120

**Published:** 2025-09-11

**Authors:** Yutao Chen, Desheng Chen, Zhixing Liang, Haoyuan Yu, Haobin Sun, Yongwei Hu, Peng Jiang, Mingshen Zhang, Linsen Ye, Hua Li

**Affiliations:** ^1^Department of Hepatic Surgery and Liver Transplantation Center of the Third Affiliated Hospital of Sun Yat-sen University, Organ Transplantation Research Center of Guangdong Province, Guangdong Province Engineering Laboratory for Transplantation Medicine, Guangzhou 510630, Guangdong, China.; ^2^Guangdong Provincial Key Laboratory of Liver Disease Research, Third Affiliated Hospital of Sun Yat-sen University, Guangzhou 510630, Guangdong, China.

**Keywords:** Hepatocellular carcinoma, immune checkpoint inhibitors, resistance, liver transplantation, therapeutic strategy

## Abstract

Hepatocellular carcinoma (HCC) poses a significant clinical burden due to its aggressive nature, profound tumor heterogeneity, and limited therapeutic efficacy. While immune checkpoint inhibitors (ICIs) have revolutionized treatment paradigms and demonstrated considerable promise, the emergence of resistance mechanisms has posed a critical challenge in contemporary clinical oncology. The accelerated development of novel agents and innovative combination strategies has further complicated this resistance landscape. In this review, we present a unique and comprehensive analysis of ICI resistance mechanisms in HCC by integrating insights into primary resistance, acquired resistance, and host-related factors. Building upon this mechanistic framework, we explore emerging therapeutic strategies to overcome ICI resistance. Furthermore, we evaluate the dual role of ICIs in HCC management - serving as a neoadjuvant therapy for transplant candidates while simultaneously posing risks of post-transplant rejection. By bridging preclinical discoveries with clinical realities, this analysis aims to inform rational therapeutic design and optimize immuno-oncology trials for HCC patients.

## INTRODUCTION

Hepatocellular carcinoma (HCC) is the most common type of primary liver cancer and the fourth leading cause of cancer-related mortality worldwide^[1]^. Although early-stage HCC can be clinically cured through surgical resection or traditional chemotherapy, the lack of apparent clinical symptoms in the early stages leads to more than 70% of patients being diagnosed at advanced stages, severely limiting therapeutic options^[2]^. The advent of immune checkpoint inhibitors (ICIs) has revolutionized therapeutic strategies for HCC. ICIs reactivate the antitumor immune response by blocking the interaction of inhibitory receptors on T cells with their ligands (e.g., PD-1, CTLA-4), thereby disarming the immune escape mechanism of tumor cells^[3]^. Recent studies have demonstrated the significant efficacy of ICIs in both immunotherapy^[4]^ and neoadjuvant therapy^[5]^ for HCC. However, the clinical response rate of ICIs is only 15%-30%, with persistent challenges of resistance^[6]^. Additionally, their application in liver transplant recipients faces risks of rejection, necessitating systematic exploration of molecular mechanisms and clinical translation strategies^[5]^.

Recent studies have revealed the complex mechanisms underlying HCC resistance to ICIs, including primary resistance, acquired resistance, and host-related factors. Primary resistance can be categorized into intrinsic and extrinsic resistance. Intrinsic resistance mechanisms include tumor immunogenicity deficiency, antigen presentation dysfunction, aberrant signaling pathways, and related genetic mutations. Extrinsic resistance primarily arises from the immunosuppressive effects of the tumor microenvironment (TME)^[7]^. Acquired resistance is predominantly linked to neoantigen loss, immune cell exhaustion, and the upregulation of immune checkpoints^[7]^. Host-related factors are mainly involved in gut microbiota dysbiosis, chronic viral infections, and systemic immune dysregulation^[8]^. These mechanisms are intricately intertwined, suggesting that monotherapy strategies are insufficient to overcome therapeutic bottlenecks, necessitating the development of combination therapies based on multidimensional biomarkers.

Furthermore, the role of ICIs in liver transplant candidates represents a double-edged sword. On the one hand, as neoadjuvant therapy, ICIs may increase transplant feasibility through tumor downstaging; on the other hand, their immune-activating effects could increase posttransplant allograft rejection risk. Current evidence regarding the optimal timing of ICI administration (e.g., pretransplant washout period), indication selection (e.g., tumor biological characteristics), and immunosuppressive regimen adjustments remains contentious, necessitating the development of individualized strategies grounded in mechanistic studies^[9,10]^.

This review aims to integrate fundamental research with clinical evidence, systematically dissect the multidimensional mechanisms of ICI resistance in HCC, and explore novel combination therapeutic approaches. Additionally, we critically assess the risk–benefit balance of ICIs during the perioperative period of liver transplantation (LT) to provide a theoretical framework for optimizing therapeutic decision making.

## IMMUNOLOGICAL TME IN HCC

As a prototypical inflammation-associated malignancy, HCC features a tumor immune microenvironment (TIME) that orchestrates the dynamic equilibrium between immunogenicity and tolerance, crucially governing tumorigenesis, therapeutic response, and immunotherapy sensitivity^[11]^. The TIME in HCC demonstrates remarkable complexity and plasticity, characterized by (1) coexisting dual immunogenic/tolerogenic properties; (2) immunosuppression-dominated microenvironment homeostasis; (3) spatiotemporal heterogeneity in dynamic evolution; and (4) metabolic reprogramming-mediated immunomodulation^[11]^.

Within the HCC TIME, immune cells exhibit a “double-edged sword” phenomenon: while executing antitumor functions through antigen presentation and cytotoxicity, they paradoxically facilitate immune evasion via checkpoint regulation and metabolic pathway manipulation^[12]^. CD8^+^ T cells mediate tumor killing via perforin, granzyme, and TNF-α secretion; however, their cytotoxic functions are markedly impaired under hypoxic/acidic conditions^[13]^. Notably, CD8^+^ T cell functional heterogeneity has prognostic significance: Notch1 signaling upregulation and exhausted phenotypes (PD-1^+^TIM-3^+^LAG-3^+^) correlate with immunotherapy resistance and poor outcomes^[14]^, whereas the XCL1-positive subset predicts favorable survival in virus-associated HCC^[15]^. With regard to M1/M2 polarization dynamics, M1 macrophages exert antitumor effects via IL-12/TNF-α secretion, whereas TREM2-driven M2 polarization promotes HCC progression by suppressing CD8^+^ T cell infiltration and enhancing tumor glycolysis^[16]^. NK cells mediate tumor surveillance through IFN-γ/NF-α release, while hypoxic stress via HIF-1α-dependent mechanisms inhibits their activation and upregulates inhibitory receptors such as NKG2A^[17]^. Complementary regulatory networks involving B cells, tumor-associated neutrophils, and cytokine cascades further contribute to immune modulation^[18]^.

The intrinsic immunosuppressive properties are particularly pronounced in HCC^[19]^. Tregs sustain immunosuppression via the TGF-β/IL-10 axis, and infiltration density is positively correlated with the stage of hepatoma^[20]^. MDSCs impair T cell and NK cell functions through the secretion of VEGF, arginase, and other mediators^[21]^, while synergizing with Tregs to amplify immunosuppression^[22]^. CAFs establish physical/biochemical barriers through PD-L1/PD-L2 expression and FASL-mediated T cell apoptosis^[23]^. These cellular populations interact with hepatic sinusoidal endothelial cells, stellate cells, and tumor-derived exosomes to maintain an immune-tolerant ecosystem^[24,25]^.

The heterogeneity of the TIME in HCC manifests in both temporal and spatial dimensions. Single-cell sequencing has revealed that early recurrent HCC harbors a distinct immune ecosystem characterized by expanded dendritic cell populations, reduced Tregs, and enriched CD8^+^ T cells in low-cytotoxicity states^[26]^. HCC is an “immune-cold tumor” phenotype characterized by inadequate T cell infiltration in tumor cores and stromal barrier formation in immune-excluded regions^[27]^, potentially explaining suboptimal responses to ICIs.

Moreover, metabolic reprogramming constitutes a cornerstone of TIME modulation in HCC. Tumor cells generate lactate via aerobic glycolysis (the Warburg effect), which activates STAT3 signaling to upregulate PD-L1 expression and expand immunosuppressive Tregs/MDSCs while directly inhibiting CD8^+^ T cell oxidative phosphorylation^[28]^. TK1 catalyzes the production of the glycolytic metabolite dTMP and nonenzymatically interacts with PRMT1, establishing a glycolysis–methylation coupling network that sustains immunosuppressive metabolic homeostasis^[29]^. In lipid metabolism, CERS5 drives HCC progression through the sphingolipid–autophagy axis activation^[30]^, whereas cholesterol dysregulation disrupts T cell receptor signaling via membrane lipid raft destabilization^[28]^.

Collectively, these factors shape the unique immune evasion mechanisms of HCC and influence the clinical response and prognosis of patients receiving immunotherapy.

## IMMUNE ESCAPE IN HCC

The immune escape mechanisms in HCC are categorized into the “3C” model: camouflage, coercion, and cytoprotection^[31]^.

Malignancies employ antigenic camouflage as a critical immune evasion mechanism by subverting immune effector cell recognition, with the tumor mutational burden (TMB; somatic mutations/megabase) serving as a key determinant of neoantigen diversity and immunotherapy responsiveness^[32]^. Low-TMB tumors exhibit defective antigen processing/presentation machinery, which is correlated with ICI resistance and poor prognosis^[33-35]^, whereas the suppression of immunogenic cell death (ICD) markers (e.g., ATP^[36]^, annexins^[37]^, calreticulin^[38]^) compromises APC recruitment/phagocytosis and T cell activation^[39]^. Notably, HCC-specific mechanisms involve HDAC8-mediated epigenetic silencing of the chemokine CCL4^[40]^ and extracellular matrix (ECM) remodeling, which results in the establishment of immune exclusion zones^[41,42]^. When camouflage fails, tumors activate coinhibitory programs through PD-L1 upregulation^[43,44]^, NKG2D ligand downregulation^[45]^, and inactivation of the cGAS-STING pathway^[46]^, coupled with metabolic immunosuppression via S-adenosylmethionine-induced T cell exhaustion^[47]^. Cytoprotective adaptations in HCC involve immune synapse destabilization through actin cytoskeletal remodeling^[48]^, JAK-STAT hyperactivation, which confers resistance to IFN-γ-mediated apoptosis^[49]^, and hypoxia-driven autophagy (HIF-1α-dependent), which degrades tumor antigens while expanding Tregs/MDSCs to sustain immunosuppressive niches^[50]^.

Importantly, the unique immune evasion mechanisms of HCC are shaped by its viral oncogenic context and molecular crosstalk. Chronic HBV/HCV infection drives PD-L1 upregulation via PI3K-AKT-mTOR activation while suppressing MHC-I antigen presentation through HBx-mediated ERAP1 inhibition, which is compounded by viral genome integration-induced low TMB that dampens neoantigen immunogenicity^[51]^. Studies have revealed that the TGF-β signaling-upregulated noncoding RNA HDAC2-AS2 in HBV^+^HCC targets CDK9 in CD8^+^ T cells via exosomes, compromising their function and offering a novel therapeutic target for HBV-associated HCC^[52]^. Additionally, tumor-initiating cells (TICs) in HCC drive immune escape through unique interactions with neutrophils. CD49f-high TICs recruit neutrophils and establish an immunosuppressive milieu via the CXCL2-CXCR2 axis, evading CD8^+^ T cell attack. Neutrophils also secrete CCL4 to induce the phenotypic conversion of neighboring tumor cells toward TIC-like states, facilitating immune evasion. CD155 overexpression is central to these mechanisms and represents a potential therapeutic target against TICs^[53]^. Furthermore, HCC metabolic reprogramming is tightly coupled with immunosuppression. For example, S100A10 activates the cPLA2/5-LOX axis to initiate lipid metabolic reprogramming, elevating LTB4 levels and promoting CD8^+^ T cell exhaustion in HCC tissues, thereby driving immune escape and enhancing tumor growth and migration^[54]^. Similarly, S100A9 + MDSCs activate the ERK/NF-κB pathway in HCC cells, creating a self-sustaining “ETV5-S100A9-ERK/NF-κB” loop via ETV5 upregulation, which accelerates tumor progression^[55]^. Hexokinase domain-containing protein 1 in glucose metabolism promotes immune escape by linking cytoskeletal dynamics, STAT1 activation, and PD-L1 upregulation in HCC^[56]^. Deficiency of mixed-lineage kinase domain-like proteins restricts Mg^2+^ release from the endoplasmic reticulum (ER) and mitochondrial Mg^2+^ uptake in HCC cells, inducing ER dysfunction and mitochondrial oxidative stress, collectively increasing susceptibility to parthanatos - a metabolic stress-dependent cell death pathway^[57]^.

HCC also exhibits distinct immunological features across various stages of tumor progression. Early recurrent HCC has unique immune escape characteristics, with increased proportions of CD161^+^CD8^+^ T cells in the microenvironment. These cells exhibit innate-like immunity, low cytotoxicity, and defects in clonal expansion. Concurrently, recurrent tumor cells competitively bind PD-L1 to CD80 on APCs, blocking CD28 costimulatory signaling and preventing T cell activation by APCs^[26]^. Additionally, studies indicate that immunosuppression in HCC progresses gradually and peaks at TNM stage II, unlike in most solid tumors, where it occurs early in carcinogenesis or before metastasis. Partial immune restoration is observed in TNM stage III tumors and is closely associated with increased neoantigen production^[58]^.

The HCC immune checkpoint landscape is dominated by CTLA-4, PD-1/PD-L1, and LAG-3, which mechanistically demarcate T cell regulation across distinct phases. CTLA-4 antagonizes CD28 costimulation during priming by competitively binding with high affinity to B7-1/B7-2 on APCs, while PD-1 mediates suppression during the effector phase by engaging PD-L1/PD-L2 in chronic, antigen-rich microenvironments^[59]^. LAG-3 complements this suppression by recognizing MHC class II on APCs, inducing ITIM/ITSM domain phosphorylation and thereby exacerbating T cell exhaustion^[60]^.

These multilayered evasion strategies enable HCC cells to orchestrate immune tolerance through the dual upregulation of ligands and the recruitment of stromal cells, creating an immunosuppressive niche. Precision-engineered ICIs counteract this tolerance by sterically blocking checkpoint axes, thereby reversing T cell anergy and restoring their cytotoxic potential against tumor cells.

## MECHANISMS OF RESISTANCE TO ICIS IN HCCS

Despite the transformative potential of ICIs in HCC management, ICI resistance remains a multifaceted clinical challenge, mechanistically stratified into primary resistance, acquired resistance, and host-related factors [Table 1]. The following paragraphs highlight this topic.

**Table 1:** Mechanisms of ICI resistance in HCC

	**Mechanism**	**Effect**
**Intrinsic resistance**	TMB, MSI ↓	Immune cell infiltration ↓^[63-65]^
	IRGQ	Antigen presentation via MHC-I ↓^[67]^
	FASN	MHC-I expression ↓^[71,72]^
	WNT/β-catenin signaling ↑	CCL5, Batf3 ↓, DC recruitment to TME ↓, antigen presentation ↓^[73,74]^
		NKG2D ↓, NK cell immune surveillance ↓^[75]^
	JAK1/2 mutation	T cell infiltration and IFN-γ signaling ↓^[79]^
	PTEN-STAT3 mutation	Cytotoxic T cell killing of tumor cells ↓^[80]^
	MerTK	Ferroptosis ↓, immunosuppressive TME ↓^[81]^
	MYC ↑	Immune escape ↑^[82]^
	TP53 mutation	Immune cell infiltration and function ↓^[83]^
	ARID1A mutation	IFN-γ signaling ↓^[85]^
	CDK20 ↑	MDSCs ↑^[86]^
	BIRC2 ↑	CD4^+^ T cells and CD8^+^ T cells ↓^[87]^
**Extrinsic resistance**	MDSCs ↑	NK cells ↓^[89,90]^
	TANs ↑	CD8^+^ T cells ↓^[91]^
	TAMs ↑	T cells ↓^[92,93]^
	WNT/β-catenin signaling ↑	CCL5 ↓, DC ↓, NK cells ↓^[76]^
	VEGF ↑	Induces FasL-mediated immune resistance^[94]^
	TGF-β ↑	CTLs and NK cells ↓, TAMs ↑^[95]^
	EMT	Induces tolerance^[99,100]^
	IDO-1	Alters collagen matrix composition, abnormal collagen deposition ↑^[102]^
		Tryptophan and kynurenine ↑^[103]^
**Acquired resistance**	B2M mutation	MHC-I ↓^[105-108]^
	PTEN	IFN-γ ↓^[109]^
	MANAs ↓	TMB ↓^[112]^
**Host-related factors**	Altered intestinal flora	Disturbed bile acid metabolism^[115,116]^
	HBV history	Tregs, MDSCs, TAMs ↑, formation of “cold tumors”^[118]^

↑: Increase/activation; ↓: decrease/deactivation. ICI: Immune checkpoint inhibitor; HCC: hepatocellular carcinoma; TMB: tumor mutational burden; MSI: microsatellite instability; IRGQ: immunity-related GTPase family Q protein; FASN: fatty acid synthase; MHC-I: major histocompatibility complex class I; WNT: proper noun, a class of signal proteins; CCL5: C-C motif chemokine ligand 5; DC: dendritic cell; TME: tumor microenvironment; NKG2D: natural killer group 2, member D; NK: natural killer; JAK1/2: Janus Kinase 1/2; IFN-γ: interferon-gamma; PTEN: proper noun, an important tumor suppressor gene; STAT3: proper noun, a kind of transcription factor; MYC: proper noun, a proto-oncogene; TP53: proper noun, a tumor suppressor gene; ARID1A: AT-rich interaction domain-containing protein 1A; CDK20: cyclin-dependent kinase 20; MDSCs: myeloid-derived suppressor cells; BIRC2: baculoviral IAP repeat-containing 2; TANs: tumor-associated neutrophils; TAMs: tumor-associated macrophages; VEGF: vascular endothelial growth factor; TGF-β: transforming growth factor-beta; CTLs: cytotoxic T lymphocytes; EMT: epithelial–mesenchymal transition; IDO-1: indoleamine 2,3-dioxygenase 1; B2M: β-2-microglobulin; MANAs: mutation-associated neoantigens; HBV: hepatitis B virus.

### Primary resistance

#### Intrinsic resistance

Intrinsic resistance mechanisms primarily involve tumor immunogenicity deficiency, dysfunctional antigen presentation, aberrant signaling pathways, and related genetic mutations.

Tumor immunogenicity is governed by the TMB and neoantigen diversity, where somatic mutations generate immunogenic neopeptides that are devoid of autoimmune risk^[61]^. The TMB, a metric that reflects the cancer mutation load, is positively correlated with neoantigen abundance and enhances T cell activation and immunogenicity. The TMB also serves as a biomarker for predicting the efficacy of PD-1 inhibitors^[62]^. Microsatellite instability (MSI) is associated with high TMB; however, most HCC patients exhibit low TMB and MSI levels^[63]^. Studies have revealed that HCC with a high TMB generates more neoantigens, activating antigen presentation and T cell responses to form an “immune-hot tumor” phenotype, characterized by increased infiltration of dendritic cells, Tregs, memory B cells, and CD8^+^ T cells^[64]^. In high-TMB HCC patients, the response to ICI treatment and prognosis are superior to those in low-TMB patients^[65]^. However, some studies have drawn conflicting conclusions: despite low TMB in HCC patients, no significant correlation exists between the TMB and prognosis^[66]^, suggesting that the TMB may be merely one factor influencing ICI resistance and cannot reliably predict ICI efficacy in HCC.

Antigen presentation dysfunction represents a pivotal mechanism underlying primary resistance to ICIs in HCC and is predominantly mediated through MHC-I downregulation. IRGQ functions as a novel autophagy receptor that interacts with GABARAPL2 and LC3B and undergoes autophagy-dependent lysosomal trafficking to degrade misfolded MHC-I heavy chains, thereby evading CD8^+^ T cell surveillance. IRGQ overexpression in HCC models potently suppresses MHC-I-mediated antigen presentation^[67]^. Clinically, MHC-I expression inversely correlates with tumor progression (reduced in 35% of early-stage HCC patients)^[68]^, whereas preserved MHC-I expression is associated with improved recurrence-free survival^[69]^ and a reduced tumor burden^[70]^. Mechanistically, FASN regulates MHC-I stability and membrane localization via palmitoylation, a posttranslational modification critical for immunomodulatory proteins such as PD-L1^[71,72]^.

Dysregulated oncogenic signaling critically underpins primary immune ICI resistance in HCC, wherein WNT/β-catenin activation suppresses CCL5 transcription and Batf3 depletion^[73,74]^, impairing DC recruitment and antigen presentation while downregulating NKG2D ligands to compromise NK cell surveillance^[75,76]^. Clinical evidence shows that HCC patients with β-catenin activation exhibit significantly reduced response rates to PD-1 inhibitors^[77,78]^. Concurrently, JAK1/2 inactivation disrupts IFN-γ signaling, reducing T cell infiltration^[79]^, whereas PTEN-STAT3 dysregulation promotes STAT3 nuclear translocation (prevalent in 60% of HCC patients), which inhibits T cell cytotoxicity and is correlated with poor prognosis^[80]^. MerTK orchestrates dual resistance mechanisms: ERK/SP1-mediated SLC7A11 activation suppresses ferroptosis, whereas MDSC recruitment via chemokine signaling cripples CD8^+^ T cell function, synergistically conferring resistance to PD-1/PD-L1 inhibitors^[81]^.

Oncogenic mutations in HCC orchestrate intrinsic ICI resistance through multifaceted immune evasion mechanisms. MYC amplification (50%-70% of HCC patients) drives PD-L1 overexpression and immunosuppression^[82]^, whereas TP53 mutation (~40% of HCC patients) impairs tumor suppressor function, recruits immunosuppressive cells, and remodels the TME immune landscape^[83]^. ARID1A mutations exhibit a paradoxical duality: enhancing TMB and PD-L1 expression via mismatch repair defects^[84]^ while restricting IFN-γ responses through chromatin remodeling^[85]^. Moreover, CDK20 activation recruits MDSCs to suppress autologous CD8^+^ T cells^[86]^, whereas BIRC2 overexpression inversely correlates with CD8^+^/CD4^+^ T cell infiltration, predicting reduced survival and ICI resistance^[87]^.

#### Extrinsic resistance

Extrinsic resistance in HCC is predominantly orchestrated by the immunosuppressive TME, which includes infiltration by immunosuppressive cellular constituents, activation of inhibitory signaling axes, dysregulated immune checkpoint expression, tumor cell phenotypic plasticity, and ECM-mediated immunomodulation.

The TME harbors immunosuppressive cells such as Tregs, MDSCs, TANs, and tumor-associated macrophages (TAMs). While Tregs physiologically maintain immune homeostasis through T cell suppression and cytokine modulation, their pathological accumulation in HCC paradoxically impairs antitumor immunity^[88]^. MDSC infiltration is correlated with ICI resistance, resulting in immunosuppression via cysteine depletion, arginase-1/inducible nitric oxide synthase upregulation, reactive oxygen species generation, and Treg expansion^[89]^. Clinical cohorts have demonstrated that elevated monocyte-derived MDSCs in HCC patients inhibit NK cell activity^[90]^. CRKL overexpression drives TAN recruitment through β-catenin stabilization and VEGFα/CXCL1 upregulation, with PD-L1^+^ TAN subsets suppressing CD8^+^ T cells via ROS/anti-inflammatory factor secretion^[91]^. M2-polarized TAMs potentiate HCC progression and ICI resistance through protumorigenic secretomes and T cell suppression^[92]^, whereas TAM-derived extracellular vesicles deliver the circPETH-encoded circPETH-147aa protein, which enhances tumor glycolysis via PKM2-mediated ALDOA-S36 phosphorylation and induces CD8^+^ T cell exhaustion through HuR-dependent methionine/leucine deprivation^[93]^.

Immunosuppressive signaling axis activation in HCC involves a mechanistic overlap with intrinsic resistance pathways, notably through Wnt/β-catenin-mediated CCL5 suppression, DC exclusion, and NKG2D ligand downregulation, collectively impairing NK cell functionality^[76]^. Additionally, the VEGF signaling pathway regulates neovascularization in HCC and induces immune resistance by increasing FasL expression, triggering the apoptosis of tumor-infiltrating CD8^+^ T cells^[94]^. TGF-β signaling orchestrates dual immunosuppression: inhibiting CTL/NK cytotoxicity through IFN-γ blockade and NKG2D/NKp30 surface depletion while polarizing myeloid cells toward M2-TAM phenotypes and suppressing macrophage/DC/neutrophil maturation^[95]^.

HCC exhibits compensatory upregulation of coinhibitory checkpoints beyond PD-1/PD-L1, with TIM-3 demonstrating DHHC9-mediated palmitoylation that stabilizes membrane localization on tumor-infiltrating CD8^+^ T/NK cells, driving exhaustion and correlating with poor prognosis^[96]^. The binding of TIM-3 to its ligand galectin-9 activates a complex signaling cascade that ultimately induces T cell exhaustion^[97]^. LAG-3 undergoes nondegradative ubiquitination by c-Cbl/Cbl-b E3 ligases upon activation, exposing the cryptic FSALE motif through basic residue-rich sequence–phospholipid interaction disruption to transmit immunosuppressive signals^[98]^. These checkpoints synergistically orchestrate PD-1/PD-L1 blockade resistance via combined T/NK cell exhaustion pathways.

Within the TME of HCC, tumor cells undergo adaptive epithelial–mesenchymal transition (EMT), driving ICI resistance through mechanisms centered on reshaping the TME into an immunosuppressive state and enhancing tumor invasiveness and adaptability. EMT promotes immunosuppressive TME formation by recruiting APCs, inducing tolerance, upregulating immune checkpoints, and resisting NK cell-mediated lysis^[99,100]^.

Additionally, dense collagen matrices within the ECM impede T cell infiltration. Indoleamine 2,3-dioxygenase 1 (IDO-1), a heme-containing enzyme in the ECM, disrupts immune clearance. In HCC, IDO-1 exacerbates the immunosuppressive microenvironment by modulating dense collagen matrices through multiple mechanisms^[101]^. First, IDO-1 regulates TAMs and Tregs to alter collagen synthesis/degradation, modifying the ECM composition and structure to create a TME conducive to HCC progression. Second, IDO-1 suppresses T cell and NK cell functions, enabling immune evasion. Tumor cells then secrete factors to stimulate fibroblast-mediated collagen synthesis, increasing matrix density. Moreover, IDO-1 interacts with the TGF-β and VEGF pathways to regulate collagen metabolism. TGF-β promotes collagen synthesis^[102]^, and IDO-1, which is regulated by TGF-β, synergistically enhances pathological collagen deposition. VEGF remodels ECM components during angiogenesis, collaborating with IDO-1 to foster a tumor-promoting niche. IDO-1 also induces tryptophan depletion and kynurenine accumulation in the TME, driving tumor resistance to ICIs^[103]^.

### Acquired resistance

Unlike primary resistance, acquired resistance refers to a patient who initially develops a clinical response to ICI therapy but subsequently experiences disease progression during treatment, characterized by dynamic post-treatment evolution. Since the mechanisms of acquired resistance in HCC have been poorly studied, we also report the mechanisms of acquired resistance identified in other malignancies. The mechanisms of acquired resistance in ICIs can be broadly classified into the following categories^[104]^: (1) defects in antigen-presentation mechanisms; (2) defects in the IFN-γ signaling pathway; (3) tumor-mediated immunosuppression; (4) other inhibitory checkpoints; and (5) clonal evolution and neoantigen loss.

Shared mechanisms underlie both primary and acquired ICI resistance, with a pathogenic convergence of β-2-microglobulin (B2M) loss-of-function mutations that destabilize MHC-I antigen presentation, a phenomenon recurrently observed in melanoma and lung cancer with acquired resistance^[105-108]^. Effector T cell-derived IFN-γ activates tumoricidal JAK-STAT signaling to upregulate MHC-I/PD-L1, although acquired resistance emerges via JAK1/JAK2 inactivation in melanoma models^[105]^. Concurrently, PTEN deletion drives PI3K-mediated immunosuppression through cytokine dysregulation and T cell exclusion^[109]^, whereas WNT-β-catenin activation similarly promotes resistance via DC dysfunction, Treg expansion, and attenuated T cell infiltration in melanoma^[110]^. Malignancies further exhibit compensatory upregulation of alternative checkpoints (TIM3, LAG3, and VISTA) as a pathoadaptive response to sustained ICI pressure^[107,111]^.

Clonal evolution and neoantigen loss represent unique mechanisms of acquired resistance. During immune editing, the immune system preferentially eliminates tumor cell clones with increased immunogenicity. Surviving tumor cells exhibit lower immunogenicity and lack critical neoantigen expression, which enables immune evasion during therapy and drives ICI resistance. Notably, NSCLC patients with post-ICI relapse exhibit loss of mutations encoding putative mutation-associated neoantigens (MANAs) in resistant tumor clones. Whole-exome sequencing revealed the disappearance of 7-18 MANAs in resistant clones that were present in pretreatment tumors^[112]^. Additionally, clonal evolution may generate intratumor heterogeneity (ITH)^[113]^, resulting in diminished tumor immunogenicity. Studies of multifocal HCC reveal mutational and copy number variation heterogeneity across lesions, with complex ITH and clonal evolution patterns impairing ICI efficacy^[114]^.

Although abnormalities in various immune cells are commonly observed across different types of drug-resistant environments, they play significantly distinct roles in the classification and mechanisms of drug resistance. In intrinsic resistance, they constitute pre-existing potent inhibitory barriers^[67,68,71,72]^. In extrinsic resistance, they serve as core executors of the TME by providing active protection and survival support^[99,100]^. In acquired resistance, they are key factors in the dynamic co-evolution with tumor cells, participating in immune editing and the remodeling of adaptive immune suppression^[105-108]^.

### Host-related factors

The gut microbiota modulates HCC responsiveness and resistance to ICIs. Dysbiosis disrupts bile acid metabolism (e.g., deoxycholic acid accumulation), impairing intrahepatic antitumor NKT cell recruitment^[115,116]^. Furthermore, post-ICI T cell responses correlate with the abundance of Bacteroides in the gut. CTLA-4 blockade induces mucosal colonization of Bacteroides species (e.g., *B. fragilis*), triggering TH_1_ cell hyperactivation and IL-12-dependent immune responses that enhance DC antigen presentation and proinflammatory cytokine production, thereby modulating ICI efficacy^[117]^. Most HCC patients have a history of HBV infection, where chronic inflammation expands Tregs, MDSCs, and M2 macrophages, suppresses effector T cell function, and fosters an “immune-cold tumor” phenotype^[118]^. Additionally, demographic variables, including age and sex, differentially regulate antitumor immunity through endocrine–immunologic crosstalk, potentially contributing to disparities in therapeutic outcomes^[119,120]^.

## DILEMMA OF ICIS IN LT

Currently, LT remains the most effective treatment for HCC, yet the application of ICIs in LT presents significant dilemmas and challenges.

### Contradiction between LT and ICIs

Pre-LT ICI administration presents a clinical conundrum: tumor control to preserve transplant eligibility *vs.* acute rejection after liver transplantation (LT-AR) risk. A case series of 10 PD-1 inhibitor-treated LT candidates demonstrated acute rejection (30%) manifesting as lobular necrosis requiring retransplantation^[121]^, with the treatment-transplantation interval emerging as a critical determinant - washout periods > 50 days reduced rejection rates to 16% (non-ICI historical baseline) without compromising oncologic outcomes^[122]^. These findings provide critical evidence for establishing a safe clinical window.

Similarly, post-LT use of ICIs in patients with HCC recurrence helps control tumor progression but risks disrupting immune tolerance, increasing the likelihood of graft rejection. Such rejection may shorten overall survival (OS) and progression-free survival (PFS)^[123]^. Studies indicate that 25% of LT recipients treated with ICIs develop acute rejection, with the rejection risk escalating when ICI initiation is closer to transplantation^[124]^. Furthermore, PD-1 inhibitor-treated LT recipients exhibit higher rejection rates than those receiving anti-CTLA-4 agents, suggesting differential rejection risks across ICI classes^[123]^. Moreover, the long-term immunosuppressants (e.g., tacrolimus and cyclosporine) required post-LT to prevent rejection may impair ICI antitumor efficacy by suppressing T cell function and increasing the risk of recurrence^[123]^. The immunosuppressive state also promotes the enrichment of immunosuppressive cells (e.g., Tregs and TAMs) in the TME, exacerbating therapeutic resistance^[9]^. The clinical data concerning the pre- and postoperative application of ICIs in LT patients are presented in Table 2.

**Table 2:** Clinical data on pre- and postoperative application of ICIs in LT patients

**Research type**	***N***	**ICIs before/after LT**	**ICIs before/after LT**	**Safety**
Retrospective cohort study^[125]^	83	Before	Camrelizumab (37.3%), Pembrolizumab (21.7%), Sintilimab (16.9%), Tislelizumab (13.3%), Nivolumab (6%), Atezolizumab (4.8%)	LT-AR (27.7%): fully recovered (65.2%), partially improved (8.7%), died (26.1%)
Retrospective cohort study^[126]^	6	Before	Atezolizumab + Bevacizumab (66.7%), Nivolumab + Ipilimumab (16.7%), Nivolumab (16.7%)	No patients had clinical evidence of rejection
Retrospective cohort study^[121]^	10	Before	Pembrolizumab or Camrelizumab + Lenvatinib	LT-AR (30%): died (66.7%)
Meta-analysis	91	Before	Nivolumab (49.5%), Pembrolizumab (23.1%), Atezolizumab + Bevacizumab (15.4%), Sintilimab (6.6%), Camrelizumab (4.4%), Durvalumab (1%)	LT-AR (26.4%): partially improved (83.3%), died (8.3%)
Pooled analysis	52	After	PD-1 blockade, Nivolumab, Pembrolizumab, Cemiplimab, Ipilimumab, Atezolizumab + Bevacizumab	LT-AR (28.8%): died (46.7%)

ICIs: Immune checkpoint inhibitors; LT: liver transplantation; LT-AR: liver transplantation-acute rejection; PD-1: programmed cell death protein 1.

### Recurrence of HCC after LT

**Figure 1:**
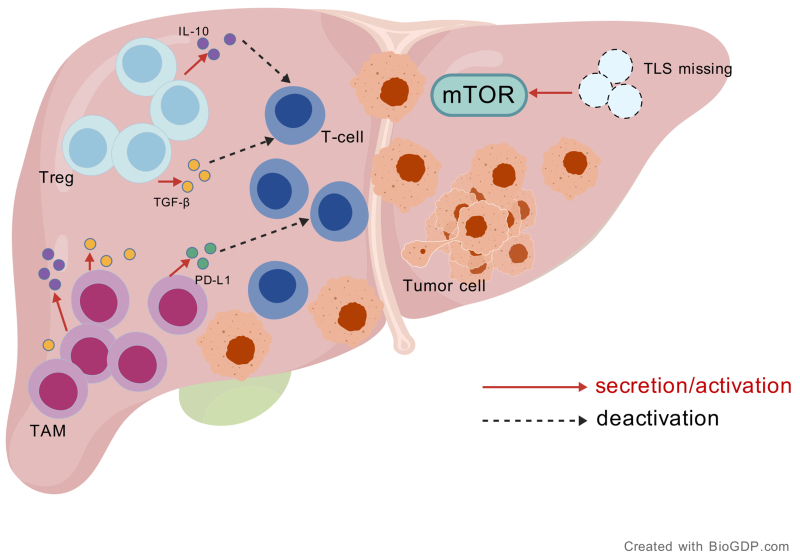
Schematic diagram of the TME associated with the recurrence of HCC after LT (created with BioGDP.com). TME: Tumor microenvironment; HCC: hepatocellular carcinoma; LT: liver transplantation.

Standard post-LT immunosuppressive regimens induce an immunotolerant niche in recurrent HCC, characterized by Treg enrichment, TAM polarization, and tertiary lymphoid structure (TLS) depletion. These agents promote Treg expansion by suppressing the CD28/CD80 axis while facilitating TAM accumulation by impairing T cell-mediated macrophage regulation. Treg/TAM-derived IL-10 and TGF-β synergistically inhibit CD8^+^ T cell cytotoxicity and upregulate PD-L1, thereby establishing an ICI-refractory microenvironment^[127]^. Concurrently, mTOR pathway activation drives TLS loss through cell cycle dysregulation, impairing antitumor immunity and reducing ICI response rates by 42% compared with those of TLS-positive counterparts^[128]^. This immunosuppressive landscape underlies the 15% post-LT HCC recurrence rate, with ICIs demonstrating limited efficacy (partial response rates < 20%) owing to adaptive TME remodeling and host immune reconstitution failure^[129]^. Studies have shown that only a subset of LT recipients with recurrent HCC achieve a partial response to nivolumab, while most exhibit treatment resistance, likely due to an altered TME and host immune adaptations [Figure 1].

### Clinical treatment dilemmas and corresponding strategies

**Figure 2:**
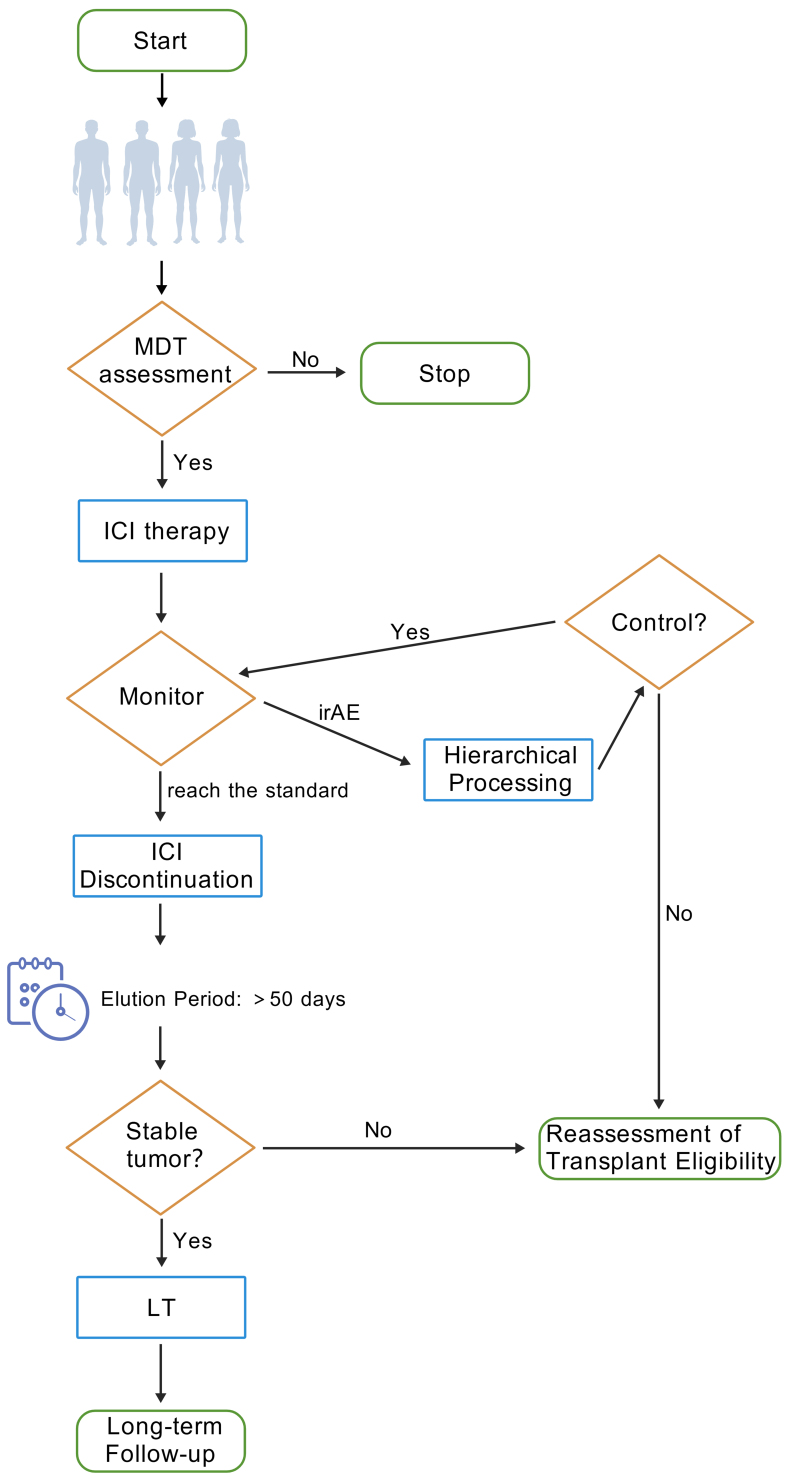
A clinical management flowchart for ICI use in LT candidates (created with BioGDP.com). ICI: Immune checkpoint inhibitor; LT: liver transplantation.

Post-LT HCC patients face multiple clinical challenges, including the lack of reliable biomarkers to predict ICI efficacy, with existing markers (e.g., PD-L1 expression, TMB) showing limited applicability under complex immunosuppressive conditions. Emerging radiomic-based quantification of TLS abundance shows promise as a noninvasive prognostic indicator for HCC-LT outcomes^[128]^, although multicenter validation is needed. The exclusion of transplant recipients from pivotal ICI trials exacerbates evidence gaps, necessitating individualized strategies such as optimized immunosuppressant titration (e.g., mTOR inhibitors such as sirolimus)^[128]^, extended pre-LT ICI washout (> 50 days)^[122]^, and graft PD-L1 profiling to guide PD-1/PD-L1 antibody selection, and PD-L1-negative grafts exhibit reduced rejection risk^[130]^. Thus, for LT recipients with recurrence refractory to other therapies, PD-L1 testing of the graft is recommended. PD-L1-negative patients may receive PD-1/PD-L1 monoclonal antibodies as salvage therapy, with close monitoring of liver function and vigilance for acute rejection. Furthermore, novel biomarkers such as TLS abundance hold promise for refining patient stratification and advancing precision therapy^[128]^. Specifically, the management process for liver transplant candidates using ICIs in clinical practice is as described in Figure 2.

ICI resistance in post-LT HCC arises from the interplay of immunosuppression, TME remodeling, and therapeutic strategy limitations. Future efforts should focus on clinical trials targeting transplant populations to develop biomarker-guided precision therapies and novel combinations while optimizing immune management to balance graft preservation and antitumor efficacy.

## TREATMENT STRATEGIES FOR HCC RESISTANT TO ICIS

### Combination therapy

#### Locoregional Therapy combined with ICIs

Locoregional therapies (LRTs), such as transarterial chemoembolization (TACE), radiofrequency ablation, and radiotherapy, induce tumor cell necrosis to release tumor-associated antigens (TAAs), increase tumor immunogenicity, activate antitumor immune responses, and increase the number of tumor-infiltrating cytotoxic CD8^+^ T cells, thereby generating synergistic effects with ICIs^[131]^. Even for recurrent HCC, LRTs combined with ICIs improve patient prognosis by remodeling the TME and reducing tumor burden^[132]^. Furthermore, novel ablation techniques such as irreversible electroporation^[133]^ and nanosecond pulsed electric fields^[134]^ disrupt tumor cell membranes to enhance antigen presentation, further optimizing the efficacy of ICIs in treating HCC.

#### Drug combination therapy

Combinatorial strategies targeting TME remodeling through multiple mechanisms are pivotal in overcoming HCC resistance to ICIs.

The atezolizumab-bevacizumab regimen, established as a first-line therapy for advanced HCC, exerts efficacy via angiogenic normalization^[135]^. Bevacizumab rectifies aberrant vasculature to enhance CD8^+^/Th-1 cell infiltration, converting immunosuppressive to immunologically active TME states, as evidenced by the IMBrave150 trial demonstrating superior OS and PFS *vs.* sorafenib (objective response rate, ORR: 30%)^[136]^. The CARES-310 trial further validated the synergy of tyrosine kinase inhibitor (TKI)-ICI, with camrelizumab-apatinib achieving a median PFS of 5.6 *vs.* 3.7 months with sorafenib in unresectable/metastatic HCC (*n* = 543), alongside an enhanced ORR and disease control rate (DCR)^[137]^. Furthermore, dual PD-1/PD-L1 and TIGIT blockade augments intratumoral cytotoxic T lymphocyte infiltration through concurrent inhibition of T cell exhaustion pathways, effectively curtailing tumor progression^[138]^.

Dual checkpoint inhibition strategies in HCC leverage synergistic targeting of complementary immune pathways. The nivolumab-ipilimumab (“O + Y”) regimen combines PD-1 blockade (alleviating PD-L1-mediated T cell suppression) with CTLA-4 inhibition (expanding T cell diversity through CTLA-4 blockade and Treg depletion), demonstrating clinical efficacy in the CheckMate-9DW trial, with a 36% ORR, 30.4-month median duration of response (DoR), and 38% 3-year OS with manageable toxicity^[139]^. This paradigm has established dual immunotherapy as a first-line HCC therapy in China.

The tremelimumab-durvalumab (“STRIDE”) regimen employs a phased approach: single high-dose CTLA-4 inhibition (tremelimumab) primes acute immune activation, whereas sustained PD-L1 blockade (durvalumab) maintains durable antitumor immunity with reduced toxicity compared with conventional dual inhibition^[140]^. TIM-3 co-targeting has also been explored. Preclinical studies have shown that TIM-3/PD-1 dual blockade in HCC mouse models significantly inhibits tumor progression, remodels the immune microenvironment, enhances T cell infiltration, and reduces PD-1/TIM-3 expression on CD8^+^ T cells, increasing ICI efficacy^[141]^.

These combinatorial approaches share a unified rationale: multidimensional modulation of angiogenesis, T cell exhaustion, and immunosuppressive cell populations to reverse TME immunosuppression, opening new therapeutic windows for resistant patients. Future efforts should prioritize biomarker-guided personalized combinations and optimize treatment sequencing to balance efficacy and safety.

### New strategies targeting drug resistance mechanisms

Emerging strategies to overcome ICI resistance in HCC focus on metabolic vulnerabilities, intrinsic resistance pathways, and extrinsic immunosuppressive networks. Metabolic targeting of TK1/LDHA and lactate accumulation reprograms immunosuppressive TMEs, with TK1 inhibitors synergizing with PD-1 blockade to enhance cytotoxic T cell infiltration and suppress tumor growth in preclinical models^[29]^, while inhibition of LPCAT1 or CERS5 restores NK cell cytotoxicity^[30]^. Spatial immune mapping via TIMES scoring can be used to predict recurrence risk and guide personalized therapy^[142]^. Intrinsic resistance mechanisms include high-affinity neoantigen (HAN)-CD39^+^CD8^+^ TIL prognostic models that optimize PD-1 inhibitor stratification^[34]^, FASN inhibition to prevent palmitoylation-mediated MHC-I lysosomal degradation^[72]^, and IRGQ-targeted autophagy modulation to restore antigen presentation^[67]^. To address signaling dysregulation, a supramolecular polypeptide that targets both β-catenin and PD-L1 has been recently developed and has demonstrated potent antitumor efficacy and biosafety in HCC immunotherapy^[143]^. Targeting MerTK with inhibitors such as sitravatinib synergizes with ICIs to induce ferroptosis in PD-L1-resistant TMEs, reduce MDSC recruitment, and activate CD8^+^ T cells^[81]^. Notably, silencing BIRC2 via shRNA or small molecules sensitizes HCC cells to immune killing, improves T cell function, and enhances ICI efficacy in preclinical models^[87]^.

With respect to extrinsic resistance, researchers have investigated several strategies. MDSC accumulation is strongly associated with ICI resistance^[86]^. CCRK overexpression in tumors promotes MDSC accumulation and is correlated with poor prognosis; CCRK inhibition reverses the immunosuppressive TME^[144]^. As CRKL contributes to the immunosuppressive TME, CRKL knockout or combined bevacizumab therapy restores anti-PD-1 efficacy in HCC mouse models, supporting the use of CRKL inhibitors plus ICIs as a promising approach^[91]^. ACE2 modulates the TME by suppressing M2-like macrophage polarization and sensitizing tumors to anti-PD-L1 therapy, highlighting the ACE2 axis as a potential immunomodulatory target^[92]^. Norathyriol, a metabolic inhibitor, reverses circPETH-147aa-driven metabolic and metastatic phenotypes, enhances CD8^+^ T cell cytotoxicity, and synergizes with ICIs to counteract metabolic immune evasion in advanced HCC^[93]^. In addition to TIM-3/PD-1 dual blockade, a peptide inhibitor targeting TIM-3 palmitoylation enhances CAR-T and NK cell antitumor activity in preclinical models, suggesting a novel therapeutic avenue for HCC^[96]^.

## CONCLUSION

Despite the demonstrated clinical benefits of ICIs in HCC, therapeutic resistance remains a major barrier to durable efficacy. This review delineates ICI applications in HCC through a tripartite framework: resistance mechanisms (intrinsic/extrinsic pathways, immunosuppressive microenvironment remodeling), LT challenges (graft rejection, immunosuppressive regimen optimization), and strategic countermeasures (biomarker-guided combinations, novel target development). Future priorities include elucidating resistance evolution via multiomics profiling, designing rational therapeutic combinations, and advancing the clinical translation of emerging modalities. Crucially, bridging mechanistic insights from preclinical models with biomarker-adaptive clinical trials will enable precision immunotherapy to balance antitumor efficacy and transplant-related immune tolerance, ultimately improving survival outcomes through vertically integrated basic–clinical research paradigms.

## DECLARATIONS

### Copyright

© The Author(s) 2025.
